# Supplementary material for: Pharmacists’ Role in Older Adults’ Medication Regimen Complexity: A Systematic Review
Source: Int J Environ Res Public Health. 2021 Aug 21;18(16):8824. doi: 10.3390/ijerph18168824 (PMC8394844; doi:10.3390/ijerph18168824)
Supplement: Supplementary file 1 [file ijerph-18-08824-s001.zip › ijerph-1308485-supplementary.pdf]

Table S1. Pubmed search strategy

1. medication regimen complexity (432)
2. treatment complexity (38803)
3. medicine complexity (29062)
4. drug complexity (16932)
5. 1 OR 2 OR 3 OR 4 (60824)
6. pharmacist (36134)
7. pharmaceutical intervention (352393)
8. pharmaceutical care (100910)
9. pharmaceutical service (83732)
10. 6 OR 7 OR 8 OR 9 (441990)
11. elderly (5203772)
12. aged (5154571)
13. older people (317120)
14. old age (232971)
15. geriatric (103867)
16. retired (24886)
17. ancient (58335)
18. 11 OR 12 OR 13 OR 14 OR 15 OR 16 OR 17 (5473335)
19. 5 AND 10 AND 18 (445)

Table S2. EPHPP quality assessment tool rating for individual studies.

STUDY DESIGN	FIRST AUTHOR	SELECTION BIAS	STUDY DESIGN	CONFOUNDERS	BLINDING	DATA COLLECTION METHODS	WITHDRAWALS AND DROP-OUTS	GLOBAL RATING
Cross-sectional	Acurcio et al. [31]	M	W	S	M	W	M	W
	Bazargan et al. [32]	W	W	S	W	S	S	W
	Lakey et al. [36]	M	W	W	M	S	M	W
	Lindquist et al. [37]	W	W	W	M	W	M	W
	Linnebur et al. [38]	M	W	W	M	S	M	W
	Pinto et al. [41]	M	W	S	W	S	M	W
	Sevilla-Sánchez et al. [43]	M	W	W	W	S	M	W
Cohort	Chang et al. [33]	M	M	S	M	S	M	S
	Elliot et al. [10]	M	M	S	M	S	M	M
	Elliot [34]	M	M	S	M	W	M	M
	Elliot et al. [12]	M	M	S	W	S	M	M
	Mansur et al. [39]	M	M	S	M	S	S	M
	Pouranayatihosseinabad et al. [42]	M	M	W	M	S	M	M
	Wimmer et al. [44]	M	M	S	S	S	S	S
	Wimmer et al. [22]	M	M	S	W	S	S	M
Quasi-experimental	Moczygemba et al. [40]	M	S	S	W	S	S	M
Prospective controlled trial	Kroenke et al. [35]	M	S	S	M	W	S	M

EPHPP= Effective Public Health Practice Project; S= strong, M= moderate, W= weak
